# Chicken meat nutritional value when feeding red palm oil, palm oil or rendered animal fat in combinations with linseed oil, rapeseed oil and two levels of selenium

**DOI:** 10.1186/1476-511X-12-69

**Published:** 2013-05-09

**Authors:** Nicole F Nyquist, Rune Rødbotten, Magny Thomassen, Anna Haug

**Affiliations:** 1 Department of Animal and Aquacultural Sciences, The Norwegian University of Life Sciences, P.O. Box 5003, Ås 1432, Norway; 2 Nofima A/S, Osloveien 1, Ås 1431, Norway

**Keywords:** Red palm oil, Palm oil, Selenium, Linseed oil, Rapeseed oil, Chicken meat, Fatty acids, Meat nutritional quality, Broiler feed

## Abstract

Chicken meat nutritional value with regard to fatty acid composition and selenium content depends on the choice of dietary oil and selenium level used in the chickens’ feed. The objective of this study was to investigate the effect of replacing commonly used rendered animal fat as a dietary source of saturated fatty acids and soybean oil as a source of unsaturated fatty acids, with palm oil and red palm oil in combinations with rapeseed oil, linseed oil and two levels of selenium enriched yeast on chicken breast meat nutritional value. The study also wished to see whether red palm oil had a cholesterol lowering effect on chicken plasma.

204 male, newly hatched broiler chickens were randomly divided into twelve dietary treatment groups, and individually fed one out of six dietary fat combinations combined with either low (0.1 mg Se /kg feed) or high (1 mg Se/kg feed) dietary selenium levels. Linseed oil, independent of accompanying dietary fat source, lead to increased levels of the *n*-*3* EPA, DPA and DHA and reduced levels of the *n*-*6* arachidonic acid (AA). The ratio between AA/EPA was reduced from 19/1 in the soybean oil dietary groups to 1.7/1 in the linseed oil dietary groups. Dietary red palm oil reduced total chicken plasma cholesterol levels. There were no differences between the dietary groups with regard to measured meat antioxidant capacity or sensory evaluation. Chicken meat selenium levels were clearly influenced by dietary selenium levels, but were not influenced by feed fatty acid composition. High dietary selenium level lead to marginally increased *n*-*3* EPA and higher meat fat % in breast muscle but did not influence the other LC PUFA levels. Chicken breast meat nutritional value from the soybean oil and low selenium dietary groups may be regarded as less beneficial compared to the breast meat from the linseed oil and high selenium dietary groups. Replacing rendered animal fat with palm oil and red palm oil had no negative effects on chicken muscle nutritional value with regard to fatty acid composition. Red palm oil decreased total chicken plasma cholesterol, confirming the cholesterol reducing effect of this dietary oil.

## Background

The consumption of preformed *n*-*3* long chained polyunsaturated fatty acids (LC PUFA) is the most efficient way of raising the level of eicosapentenoic acid (EPA), docosapentaenoic acid (DPA) and docosahexaenoic acid (DHA) in humans, as our endogenous production of these *n*-*3* LC PUFAs from 18:3*n*-*3* alpha-linolenic acid (ALA) is quantitatively limited [1-4]. Consumers are being encouraged to increase their dietary intake of *n*-*3* LC PUFA by consuming more fish or marine oil supplements. Despite general recommendations, fish consumption has not increased markedly, while the consumption of meat and meat products, including boiler meat is rising [5,6]. The utilization of the world marine resources is reaching its limitations, stagnating the availability of marine oils [7]. Resource limitations and consumer dietary preferences have lead to an increased focus on finding alternative ways of supplying dietary *n*-*3* LC PUFAs and antioxidants such as selenium (Se) to the consumer. Utilizing the capacity of the chicken to produce LC PUFAs from both 18:2*n*-*6* linoleic acid (LA) and ALA represents a good way of supplying the human consumer with preformed dietary LC PUFA [8].

Dietary levels of *n*-*6*, *n*-*3* and Se may influence the pathogenesis of inflammation and development of diseases such as cancer, Alzheimer and cardiovascular diseases, affect reproductive health and play key roles in mental and immune system development of both humans and animals [9-13]. Optimizing chicken meat nutritional value by adjusting level of Se and fatty acid composition in chicken feed is one way of influencing the overall consumption of these essential nutrients [14-17].

The positive effect of replacing *n*-*6* rich soybean oil (SO) with *n*-*3* rich linseed oil (LO) and rapeseed oil (RO) on the nutritional value of chicken meat has been documented [14-17]. Little information is, however, available on the effects of replacing the saturated fatty acid (SFA) rendered animal fat (FR), with palm oil (PO) or red palm oil (RPO).

Unrefined RPO is naturally rich in antioxidants [18] and, in comparison to other dietary oils rich in the SFA 16:0, has been shown to reduce plasma cholesterol levels [19,20]. As fatty acid synthesis and cholesterol biosynthesis are comparable in humans and chickens [21], plasma cholesterol levels in chickens will be included in the current study to see the effect of RPO on chicken plasma cholesterol levels.

In this context, our objectives were to investigate chicken breast muscle nutritional value and sensoric qualities, with respect to fatty acid composition and total Se levels, when replacing the SFA source FR in chicken feed with PO and RPO in combinations with LO and RO and two levels of Se enriched yeast.

## Methods

### Diets

Twelve wheat based meal feeds, identical in composition with the exception of dietary oil source and level of Se, were used in the experiment (Table 1).

**Table 1:** Composition of the experimental diets

**Diet**	**1**	**2**	**3**	**4**	**5**	**6**	**7**	**8**	**9**	**10**	**11**	**12**
**Ingrediens (%)**	**FR + SO**	**FR + LO**	**PO + LO**	**RPO + LO**	**FR + LO + RO**	**PO + LO + RO**	**FR + SO**	**FR + LO**	**PO + LO**	**RPO + LO**	**FR + LO + RO**	**PO + LO + RO**
Wheat	45	45	45	45	45	45	45	45	45	45	45	45
Corn gluten	10	10	10	10	10	10	10	10	10	10	10	10
Soybean flour	17	17	17	17	17	17	17	17	17	17	17	17
Oat	15	15	15	15	15	15	15	15	15	15	15	15
Rendered-fat (FR)	4	5.6	-	-	4	-	4	5.6	-	-	4	-
Soybean oil (SO)	4	-	-	-	-	-	4	-	-	-	-	-
Refined palm oil (PO)	-	-	5.6	-	-	4	-	-	5.6	-	-	4
Red palm oil (RPO)	-	-	-	5.6	-	-	-	-	-	5.6	-	-
Rapeseed oil (RO)	-	-	-	-	1.6	1.6	-	-	-	-	1.6	1.6
Linseed oil (LO)	-	2.4	2.4	2.4	2.4	2.4	-	2.4	2.4	2.4	2.4	2.4
Se yeast *	0.003	0.003	0.003	0.003	0.003	0.003	0.04	0.04	0.04	0.04	0.04	0.04
Minor constituents**	5	5	5	5	5	5	5	5	5	5	5	5

*Organic Se yeast (Bio-Logics Inc. New O.S.Y 2000X) containing 2.15 g Se per kg.

**Minor constituents of feed: Histidine 0.1% , choline chloride 0.13%, mono-calcium phosphate 1.4%, ground limestone 1.3%, sodium chloride 0.25%, sodium bicarbonate 0.2%, vitamin A 0.03%, vitamin E 0.06%, vitamin ADKB 0.09%, vitamin D3 0.08%, L-lysine 0.4%, DL-methionine 0.2%, L-threonine 0.2%.

The wheat grain in the meal was ground in a hammer mill with a five-millimeter sieve. Six different oil blends and two levels of organic Se were used to formulate the 12 diets. The oils used were rendered animal fat (Norsk Protein AS, Smiuhagan, Ingeberg, Norway), soybean oil (Felleskjøpet Agri, Noway.), linseed oil (Leinöl native. Naturata AG. D-71711 Murr), palm oil (Fritex 35, AarhusKarlshamn, Sweden AB), red palm oil (Ruker Palm oil, Ruker Ventures LTD, Ghana, West Africa) and rapeseed oil (Odelia cold pressed Rapeseed oil, Askim Bær- og Fruktpresseri, Askim, Norway). Organic Se enriched yeast (BioLogics, Ultra Bio-Logics Inc. New O.S.Y. 2000X, containing 2.15 g Se/kg) was included at low (0.003% Se enriched yeast, giving 0.065 mg Se/kg feed and a total Se in feed of 0.13 mg Se/kg diet) (low Se) and high (0.04% Se enriched yeast, giving 0.86 mg Se/kg feed and a total Se in feed of 1.05 mg Se/kg diet) (high Se) levels. The dry ingredients were weighed and mixed (Forberg twin-shaft paddle mixer, F-60) prior to adding the oils by spraying (VeeJet flat spray nozzle, spraying systems Co). After mixing, the diets were packed in 20 kg, light proof paper sacks and stored at room temperature during the trial. The trial started the day after the feed had been produced. The feeds were produced at FôrTek, 1432 Ås, Norway.

### Animals and housing

The animals used in this experiment were treated in accordance with national and international guidelines concerning the use of animals in research (Norwegian Animal Welfare Act, European Convention for the Protection of Vertebrate Animals used for Experimental and Other Scientific Purposes, CETS No.: 1# 1986). The animals were inspected twice daily by qualified handlers, and every other day by a veterinarian throughout the trial period. The study was approved by the National Animal Research Authority representative at the Norwegian University of Life Sciences.

250 newly hatched Ross 308 chickens (Nortura Samvirkekylling, Norway), were randomly divided into 12 groups. Each group was collectively weighed, and placed in mesh floored, battery cages. Each group received one of the 12 diets from day one until four weeks of age. On day 12 each group was collectively weighed before each bird was weighed individually. 17 chickens from each group were selected and placed in separate metabolism cages ordered randomly in one of two rooms, resulting in a total of 204 chickens. The birds were individually fed from day 12 onwards. The chickens were individually weighed again on days 20 and at trial termination. The cages were kept in environmentally controlled rooms, where the temperature was maintained at 32°C for the first three days, then reduced gradually by 0.5°C per day until reaching 21°C on day 21. This temperature was held for the rest of the period. The light regime was set to 24 hours light for the first day, followed by six days with 23 hours light and one hour of darkness. From day seven the lights were turned off for two four-hour periods per day, 00–04 h and 17–21 h. The chickens had free access to feed and water throughout the experiment. General health and mortality rates were registered daily.

### Sampling

For sampling, chickens were stunned by a sharp blow to the head and killed by exsanguination. Blood was collected from all individuals using heparinized blood collection vials (Li-heparin Vacutainer® blood collection vials, BD Norge AS, Trondheim, Norway). Blood was sentrifuged at 1000 × G for 15 minutes before palsma being stored at – 20°C for later analysis.

After slaughtering the chickens right breast muscle was removed, vacuum packed and stored at -20°C for six months before sensory evaluation. From the left breast muscle, caudal to cranial, samples were taken for antiradical power (ARP) analysis, total Se analysis and fatty acid analysis. All samples were individually packed and stored at - 20°C.

### Analyses

An analysis of plasma cholesterol was carried out at the Central laboratory at the Norwegian School of Veterinary Science according to standard procedures.

Fatty acid composition of breast muscle and feed was determined by gas chromatography. Lipid extraction and direct methylation were performed in accordance with O’Fallon et al. [22]. The fatty acid methyl esters (FAME) were subsequently separated by a fused silisiumdioksid capillary column (200 m × 0.25 mm i.d. × 0.25 μm film thickness).

The analysis of Se content was performed according to NMKL method 161. The analyses were conducted by Eurofins AS, Moss, Norway, who utilize a ICP-AES instrument (Perkin Elmer, Optima 7300). Prior to analysis the samples were pretreated by addition of HNO_3_ and H_2_O_2_ and digested in a laboratory microwave oven for 20 min. up to 180°C. Limit of quantification (LOQ) was 0.05 mg/kg.

The antioxidant activity of chicken breast muscle was determined by using the free radical 2,2-diphenyl-1-picrylhydrazyl (DPPH), according to the procedure described by Brand-Williams et al. (1995) and the antioxidant activity of breast meat is given as the reciprocal of EC50, the antiradical power in units of mg of DPPH per g meat as described by Mielnik et al. (2003) [23,24].

### Sensory evaluation

Descriptive sensory analysis (ISO 6564:1985E and ISO 13299:2003E) was performed with a trained sensory test panel, consisting of 11 people, that assessed 18 sensory traits within smell, taste and texture and gave the grades one-nine, one being no intensity and nine clear intensity of the tested trait parameter. The individually vacuum-packed, frozen chicken breast fillets were thawed and divided longitudinally to produce two samples. The samples were placed in plastic bags, labelled and vacuum packed. The samples were prepared by placing them on a grid and heat treated with steam at 80°C for eight minutes, and then served to the judges. The samples were randomly served according to feed group, judge and repetition.

### Statistical analysis

Data from each chicken, housed in individual metabolism cages, served as the experimental unit. Results are presented as least square means of the twelve dietary groups. Statistical analyses, apart from sensory data, in this study were done by the “Statistical Analysis System”, SAS 9.1 ANOVA using General Linear Model procedure and Ryan-Einot-Gabriel-Welsch Multiple Range Test to establish statistical significant differences between the parameters of the twelve dietary groups. SAS (Proc GLM) two-way ANOVA was performed with oil and Se level as class variables and Ryan-Einot-Gabriel-Welsch range test for comparisons of main effects. Results were regarded as significant when *P* < 0.05. Sensory data were analyzed by SAS 9.1.3 ANOVA variance analysis. Where the F-tests showed significant differences an additional Turkeys test was performed to identify which samples that were different.

## Results

### Fatty acid composition

Fatty acid composition of the 12 diets and the breast meat from the 12 dietary groups, are presented in Tables 2, 3 and 4.

**Table 2:** Fatty acid composition of experimental diets

**Diet**	**1**	**2**	**3**	**4**	**5**	**6**	**7**	**8**	**9**	**10**	**11**	**12**
**Oil**	**FR + SO**	**FR + LO**	**PO + LO**	**RPO + LO**	**FR + LO + RO**	**PO + LO + RO**	**FR + SO**	**FR + LO**	**PO + LO**	**RPO + LO**	**FR + LO + RO**	**PO + LO + RO**
**Se level**	**Low**	**Low**	**Low**	**Low**	**Low**	**Low**	**High**	**High**	**High**	**High**	**High**	**High**
14:0	1.0	1.3	0.6	0.6	1.0	0.5	1.0	1.3	0.6	0.6	1.0	0.5
16:0	17.5	18.2	28.1	28.9	15.3	22.4	17.4	18.1	28.0	28.5	15.3	22.2
18:0	8.2	10.2	3.4	3.5	7.9	2.9	8.0	10.1	3.3	3.5	7.9	2.9
16:1n-9	1.2	1.6	0.2	0.2	1.2	0.2	1.2	1.6	0.2	0.2	1.2	0.2
18:1n-9	27.1	28.1	30.3	29.4	31.5	32.9	26.9	28.1	30.2	29.8	31.5	32.9
18:2n-6	35.2	19.7	21.7	21.7	21.4	23.3	35.7	19.8	21.8	21.7	21.4	23.2
18:3n-3	4.0	14.2	13.5	13.4	15.8	15.2	4.0	14.3	13.6	13.5	15.8	15.5
LA/ALA	8.8	1.4	1.6	1.6	1.4	1.5	8.9	1.4	1.6	1.6	1.4	1.5
SFA*	26.6	29.7	32.0	33.0	24.2	25.8	26.4	29.6	31.9	32.6	24.2	25.6
MUFA**	28.2	29.7	30.4	29.6	32.7	33.0	28.1	29.7	30.3	29.9	32.7	33.1
PUFA***	39.2	33.9	35.2	35.2	37.2	38.4	39.7	34.1	35.4	35.2	37.3	38.6

Linoleic acid (LA) alpha-linolenic acid (ALA), *SFA: 14:0, 16:0, 18:0, **MUFA: 16:1n-9, 18:1n-9, ***PUFA: 18:2n-6, 18:3n-3.

g/100 g fatty acid methyl ester (% FAME).

**Table 3:** Breast muscle fatty acid profile mg/g wet weight muscle

**Oil**	**FR + SO**	**FR + LO**	**PO + LO**	**RPO + LO**	**FR + LO + RO**	**PO + LO + RO**	**FR + SO**	**FR + LO**	**PO + LO**	**RPO + LO**	**FR + LO + RO**	**PO + LO + RO**	**P model**
**Se level**	**Low**	**Low**	**Low**	**Low**	**Low**	**Low**	**High**	**High**	**High**	**High**	**High**	**High**	
N	17	17	16	17	17	16	16	16	16	17	17	17	
16:0	1.63	1.41	1.90	1.79	1.61	1.83	2.07	1.61	1.91	2.01	1.96	2.04	0.0445
16:1n-9	0.16	0.15	0.16	0.15	0.19	0.15	0.24	0.20	0.18	0.18	0.27	0.18	0.1303
18:0	0.96abc	0.90bc	0.82c	0.81c	0.96abc	0.83c	1.11a	0.95abc	0.80c	0.83c	1.09ab	0.88c	<.0001
18:1n-9 Oleic acid	2.05ab	1.89b	2.31ab	2.08ab	2.55ab	2.34ab	2.77ab	2.27ab	2.36ab	2.51ab	3.34a	2.77ab	0.0562
18:2n-6 LA	1.90ab	1.10c	1.35bc	1.32bc	1.40bc	1.45bc	2.48a	1.25bc	1.43bc	1.47bc	1.72bc	1.61bc	<.0001
18:3n-3 ALA	0.12d	0.37bcd	0.45bc	0.41bcd	0.58ab	0.51b	0.17cd	0.48bc	0.48bc	0.52b	0.82a	0.65ab	<.0001
20:4n-6 AA	0.62a	0.30b	0.31b	0.32b	0.30b	0.31b	0.62a	0.30b	0.31b	0.31b	0.31b	0.32b	<.0001
20:5n-3 EPA	0.03c	0.19ab	0.18ab	0.18ab	0.18ab	0.17b	0.03c	0.20a	0.18ab	0.18ab	0.18ab	0.19ab	<.0001
22:5n-3 DPA	0.14c	0.31ab	0.31ab	0.32ab	0.32ab	0.30ab	0.13c	0.31ab	0.28b	0.31ab	0.33a	0.31ab	<.0001
22:6n-3 DHA	0.14cd	0.21a	0.19ab	0.20a	0.19ab	0.20ab	0.12d	0.18ab	0.16bc	0.20ab	0.20ab	0.19ab	<.0001
Sum SFA^I^	2.67	2.39	2.78	2.66	2.66	2.71	3.31	2.66	2.77	2.90	3.18	2.98	0.1602
Sum MUFA^II^	2.26ab	2.10b	2.50ab	2.25ab	2.81ab	2.52ab	3.08ab	2.54ab	2.57ab	2.71ab	3.69a	2.98ab	0.0639
Sum PUFA^III^	3.40ab	2.88b	3.18ab	3.13ab	3.41ab	3.36ab	4.05a	3.13ab	3.22ab	3.39ab	4.06a	3.68ab	0.0078
Sum all FA	8.34	7.38	8.46	8.04	8.88	8.59	10.44	8.32	8.55	8.99	10.92	9.64	0.0677
Sum n-3 LC PUFA^IV^	0.31c	0.71a	0.68ab	0.70a	0.68ab	0.67ab	0.29c	0.68ab	0.62b	0.68ab	0.71a	0.69ab	<.0001
n-6:n-3^V^	5.85b	1.30c	1.48c	1.49c	1.37c	1.49c	6.54a	1.34c	1.60c	1.50c	1.34c	1.46c	<.0001
LA:ALA	17.23a	3.24b	3.23b	3.69b	2.82b	3.12b	17.37a	3.01b	3.59b	3.21b	2.43b	2.81b	<.0001
AA:EPA	19.04a	1.59b	1.76b	1.84b	1.66b	1.86b	19.05a	1.54b	1.73b	1.81b	1.71b	1.74b	<.0001
AA:n-3LCPUFA	2.03b	0.42c	0.46c	0.47c	0.44c	0.47c	2.20a	0.44c	0.50c	0.46c	0.44c	0.46c	<.0001

Statistically significant differences between groups are indicated by differing superscript letters a-d (P < 0.05, REGW multiple range test).

^I^SFA: 14:0, 15:0, 16:0, 17:0, 18:0, 20:0.

^II^MUFA: 14:1n-9, 16:1n-9, 18:1t6n-11, 18:1n-9.

^III^PUFA: 18:2n-11, 18:2n-6, 18.3n-6, 18:3n-3, 20:2n-6, 20:3n-6, 20:3n-3, 20:4n-6, 20:5n-3, 22:5n-3, 22:6n-3.

^IV^n-3 LC PUFA: EPA + DPA + DHA.

^V^n-6: LA + AA , n-3: ALA + EPA + DPA + + DHA.

**Table 4:** Breast muscle fatty acid profile mg/g wet weight muscle

	**Oil**						**Se level**				
	**FR** + **SO**	**FR** + **LO**	**PO** + **LO**	**RPO** + **LO**	**FR** + **LO** + **RO**	**PO** + **LO** + **RO**					
	**n = 32**	**n = 33**	**n = 32**	**n = 34**	**n = 34**	**n = 33**	**Low**	**High**	**P (Oil source)**	**P (Se)**	P (Oil source*Se)
16:0	1.84	1.50	1.91	1.90	1.79	1.94	1.69b	1.94a	0.0619	0.0070	0.8080
16:1n-9	0.20	0.17	0.17	0.16	0.23	0.16	0.16b	0.21a	0.1661	0.0088	0.8685
18:0	1.03a	0.93ab	0.81b	0.82b	1.03a	0.85b	0.88b	0.94a	<.0001	0.0223	0.3650
18:1n-9, Oleic acid	2.39ab	2.07b	2.33ab	2.29ab	2.94a	2.56ab	2.20b	2.67a	0.0778	0.0070	0.8406
18:2n-6 LA	2.17a	1.17b	1.39b	1.40b	1.56b	1.53b	1.42b	1.65a	<.0001	0.0089	0.6400
18:3n-3 ALA	0.15c	0.42b	0.47b	0.47b	0.70a	0.58ab	0.41b	0.53a	<.0001	0.0056	0.6982
20:4n-6 AA	0.62a	0.30b	0.31b	0.32b	0.31b	0.32b	0.36	0.36	<.0001	0.8663	0.8111
20:5n-3 EPA	0.03c	0.19a	0.18b	0.18b	0.18ab	0.18b	0.16b	0.16a	<.0001	0.1997	0.4032
22:5n-3 DPA	0.14c	0.31ab	0.30b	0.31ab	0.32a	0.31ab	0.28	0.28	<.0001	0.4601	0.2163
22:6n-3 DHA	0.13b	0.19a	0.18a	0.20a	0.19a	0.20a	0.19	0.18	<.0001	0.0318	0.2189
Sum SFA^I^	2.97	2.52	2.77	2.78	2.92	2.85	2.64b	2.96a	0.3162	0.0085	0.7112
Sum MUFA^II^	2.64	2.31	2.54	2.48	3.25	2.76	2.41b	2.93a	0.0890	0.0071	0.8448
Sum PUFA^III^	3.71a	3.00b	3.20ab	3.26ab	3.73a	3.53ab	3.23b	3.59a	0.0061	0.0077	0.7389
Sum n3-LC PUFA^IV^	0.30b	0.70a	0.65a	0.69a	0.70a	0.68a	0.62	0.62	<.0001	0.2208	0.1220
Sum all FA	9.32	7.83	8.51	8.52	9.90	9.13	8.28b	9.48a	0.1025	0.0072	0.7818
AA:EPA	19.05a	1.57b	1.75b	1.82b	1.68b	1.80b	4.68	4.36	<.0001	0.8936	0.9999

Two-way ANOVA with oil and Se level as class variables. Statistically significant differences between groups are indicated by differing superscript letters a-b (P < 0.05, REGW multiple range test).

^I^SFA: 14:0, 15:0, 16:0, 17:0, 18:0, 20:0.

^II^MUFA: 14:1n-9, 16:1n-9, 18:1t6n-11, 18:1n-9.

^III^PUFA: 18:2n-11, 18:2n-6, 18.3n-6, 18:3n-3, 20:2n-6, 20:3n-6, 20:3n-3, 20:4n-6, 20:5n-3, 22:5n-3, 22:6n-3.

^IV^n-3 LC PUFA: EPA + DPA + DHA.

Chickens in the SO containing groups had significantly higher levels of *n*-*6* LA, while the FR + LO dietary groups had the lowest levels of *n*-*6* LA in breast muscle. Alpha-linolenic acid content was approximately three times higher for the dietary groups containing both LO and RO, and lowest for the SO containing groups. These values were reflected in the LA:ALA ratios as the SO based groups had the highest LA:ALA ratio compared to the LO containing dietary groups, independent of which dietary oils LO was combined with. The ratio between AA: EPA was roughly ten times higher for the SO dietary groups. The sum of PUFA was highest for the SO and the FR+LO+RO dietary groups, while the FR + LO diet resulted in the lowest PUFA values in breast muscle. For the SO dietary groups the *n*-*6* PUFA dominated compared to the LO dietary groups where the *n*-*3* PUFA levels made up the bulk of the PUFA.

For the LC PUFAs there were clear differences between the SO and the LO containing dietary groups breast meat fatty acid compositions. The AA levels were almost twice as high in SO groups’ breast meat compared to the rest of the dietary groups. The amounts of EPA, DPA and DHA found in the SO dietary groups were lower compared to the LO dietary groups. This difference was reflected in the sum of *n*-*3* LC PUFAs found in the chicken breast meat, as the LO feed groups all had significantly higher levels of these fatty acids when compared to the SO groups.

As seen in Table 4 the dietary Se level influenced the total level of fat in chicken breast muscle, the highest level of Se leading to the highest level of fat. The higher level of fat is reflected in higher levels of saturated fatty acids (SFA), monounsaturated fatty acids (MUFA) and PUFA for the high Se groups. The dietary Se concentration only marginally influenced level of EPA found in the chickens’ breast muscles, but had no affect on the other LC PUFA levels.

### Total selenium, plasma cholesterol levels and antiradical power

Table 5 shows total Se (mg/kg muscle) in chicken breast meat, and total plasma cholesterol levels (mmol/L). The total Se in breast muscle was as anticipated lowest for the low Se and highest for the high Se dietary groups, independent of oil source.

**Table 5:** Total Se in chicken muscle and feed and total plasma cholesterol levels in chicken

**Oil**	**FR + SO**	**FR + LO**	**PO + LO**	**RPO + LO**	**FR + LO + RO**	**PO + LO + RO**	**FR + SO**	**FR + LO**	**PO + LO**	**RPO + LO**	**FR + LO + RO**	**PO + LO + RO**	**P**
**Selenium level**	**Low**	**Low**	**Low**	**Low**	**Low**	**Low**	**High**	**High**	**High**	**High**	**High**	**High**	**model**
**N**	**17**	**17**	**16**	**17**	**17**	**16**	**16**	**16**	**16**	**17**	**17**	**17**	
Total Se in muscle (mg/kg)	0.09b	0.09b	0.09b	0.10b	0.09b	0.14b	0.60a	0.56a	0.58a	0.59a	0.57a	0.59a	0.0001
Total Se in feed (mg/kg)	0.15	0.13	0.14	0.14	0.12	0.12	1.2	0.99	1.1	1.0	0.99	1.0	
Cholesterol (mmol/L)	4.09 a	4.12a	4.02a	**3.81b**	4.19a	3.96ab	4.17a	4.06a	4.13a	**3.84b**	4.09a	4.06a	0.0252

Statistically significant differences between groups are indicated by differing superscript letters a-b (P < 0.05, REGW multiple range test).

Total plasma cholesterol levels were lowest for the RPO dietary groups, and were not influenced by the dietary Se level. Total plasma cholesterol did not differ between the other dietary oil groups.

Chicken breast muscle antiradical power, expressed as mg/g DPPH, did not differ between the dietary groups independent of dietary Se level and oil source.

### Sensoric evaluation

The sensoric evaluation for the breast muscle showed no significant differences among the groups when it came to taste and texture after six months storage at -20°C (Table 6). There were, however, some differences when it came to odor. The high Se RPO + LO dietary group had the highest intensity for acidulous odor, and lowest intensity for stale odor, while the high Se FR + LO + RO group had the highest intensity for stale odor. Acidulous taste and smell relates to a fresh, sweet and sour experience while a stale taste and smell relates to an unfresh, low aromatic, nauseous or oversweet experience.

**Table 6:** Sensory evaluation of chicken breast muscle after storage for six months at -20°C

**Oil**	**FR + SO**	**FR + LO**	**PO + LO**	**RPO + LO**	**FR + LO + RO**	**PO + LO + RO**	**FR + SO**	**FR + LO**	**PO + LO**	**RPO + LO**	**FR + LO + RO**	**PO + LO + RO**	**P model**
**Se level**	**Low**	**Low**	**Low**	**Low**	**Low**	**Low**	**High**	**High**	**High**	**High**	**High**	**High**	
**N**	**17**	**17**	**16**	**17**	**17**	**16**	**16**	**16**	**16**	**17**	**17**	**17**	
**Flavor:**													
Acidulous	3.0	2.7	2.8	3.0	2.6	3.2	3.2	3.3	3.0	3.1	2.7	2.8	0.34
Sweet	2.8	2.9	2.8	2.8	2.7	2.7	2.6	2.8	2.8	2.8	2.7	2.8	0.94
Salty	2.2	2.2	2.2	2.2	2.3	2.2	2.3	2.3	2.3	2.3	2.2	2.2	0.91
Metallic	5.4	5.1	4.9	5.3	4.7	5.1	5.4	5.2	5.0	5.1	5.1	5.2	0.56
Bitterness	4.3	4.6	4.5	4.3	4.6	4.3	4.2	4.4	4.5	4.5	4.5	4.7	0.68
Plant oil	1.5	1.6	1.4	1.5	1.8	1.6	1.5	1.6	1.5	1.6	1.6	1.7	0.92
Rancid	1.7	1.6	1.6	1.4	2.2	1.6	1.7	1.7	1.6	1.6	2.0	1.8	0.30
Stale	2.5	3.2	3.0	2.7	3.1	2.7	2.7	2.8	3.2	2.6	3.3	3.0	0.09
**Odor:**													
Acidulous*	2.7a	2.5a	2.7a	2.9a	3.0a	3.0a	2.6a	3.1a	2.6a	3.2a^*^	2.5a	2.5a	0.01
Sweet	2.8	2.8	2.9	2.9	3.0	2.9	3.0	2.9	2.8	3.2	3.0	2.8	0.49
Metallic	4.2	4.0	4.1	4.2	4.2	4.1	4.2	4.2	4.0	3.9	4.0	4.2	0.90
Plant oil	1.2	1.5	1.4	1.4	1.6	1.3	1.5	1.3	1.3	1.3	1.6	1.6	0.74
Rancid	1.5	1.8	1.4	1.1	1.5	1.4	1.5	1.3	1.5	1.1	1.5	1.8	0.13
Stale	3.1ab	3.6ab	2.9ab	2.9ab	3.1ab	3.0ab	3.2ab	3.0ab	3.4ab	2.7b	3.7a	3.5ab	0.01
**Texture:**													
Hard	3.8	4.4	4.1	4.2	4.3	4.4	4.3	4.3	4.5	4.1	4.6	4.2	0.16
Tenderness	5.5	5.0	5.1	5.1	5.0	5.0	5.0	5.1	4.7	5.2	4.9	5.0	0.59
Fatty	2.7	2.7	2.5	2.6	2.8	2.6	2.7	2.7	2.7	2.7	2.7	2.7	0.72
Juicy	4.4	4.3	4.1	4.1	4.4	4.3	4.3	4.5	4.3	4.4	4.5	4.3	0.88

Statistically significant differences between groups are indicated by differing superscript letters a-b, with Turkeys test (P < 0, 05).

* Turkeys test was not able to identify where the difference lay but the High Se RPO + LO group had the highest intensity for acidulous smell.

## Discussion

In the current study the effect of varying chicken dietary saturated and unsaturated fat source and Se level on breast meat nutritional value, sensoric evaluation and chicken plasma cholesterol levels were studied.

The breast meat fatty acid composition of the 12 dietary groups may to a large extent be attributed to the composition of the dietary oils used in the respective diets. These results are in agreement with earlier studies showing clear correlations between dietary fatty acid composition and the fatty acid composition of chicken meat [25].

The greatest difference in *n*-*6* and *n*-*3* fatty acid composition was seen between the chickens consuming the SO based diets compared to the LO based diets, regardless of which SFA source the LO was paired with. In agreement with earlier studies, the LO dietary groups all showed higher levels of ALA, EPA, DPA and DHA when compared to the SO dietary groups which, on the other hand, had the highest level of both LA and AA [17,26-28]. Dietary ALA and LA levels were mirrored both by their levels in chicken breast meat, and by the levels of their 20 carbon fatty acids metabolites EPA and AA, respectively [29]. The ratio between LA and ALA was almost six times lower for the LO dietary groups compared to the SO dietary groups. The ratio between AA and EPA was reduced more than ten times, from 19:1 in the SO dietary groups, to about 1.7/1 in the LO dietary groups. Increasing the dietary level of *n*-*3* LC PUFA and lowering AA:EPA ratio will reduce the amount of AA in cell membranes available for pro-inflammatory eicosanoid production, suppressing the development and severity of many common chronic diseases such as cardiovascular diseases, cancer, inflammatory and autoimmune diseases [13,27,30,31]. Chicken meat nutritional value from the SO dietary groups, containing less of the beneficial *n*-*3* EPA and DHA, higher levels of AA and considerably higher LA:ALA ratio, may be regarded as less healthy compared to the breast meat from the LO dietary groups. Consuming chicken meat enriched in *n*-*3* LC PUFA may positively influence the level of EPA in human serum phospholipids [32].

Consuming a 175 g portion of chicken breast meat [33] from the current study LO supplemented dietary groups, would supply the consumer with about 119 mg of preformed EPA + DPA + DHA, compared to 53 mg if consuming the meat from the SO dietary groups. The proposed European Food Safety Authority (EFSA) reference intake value for *n-3* LC PUFA to reduce the risk of cardiovascular disease is 250 mg/day [34].

Earlier studies on dietary Se levels and PUFA in chickens [16,17] have reported increased muscle *n-3* LC PUFA levels when increasing dietary Se levels. In the current study the high Se dietary groups only showed a marginal increase in the level of EPA, but failed to show any affect on the other LC PUFA. In a study by Kralik et al. (2012) a dietary Se level of 0.3 mg/kg feed lead to higher ALA, EPA, DPA and DHA levels, while a dietary Se level of 0.5 mg/kg did not significantly increase the levels of these fatty acids when compared to a non Se supplemented diet [35]. The inclusion of 0.07 mg Se/kg feed from selenium enriched yeast in the low Se diets, resulted in the total Se content of feed being 0.13 mg Se/ kg, which is below The National Research Council (NRC, 1994) recommended 0.15 mg Se/kg diet, but may still need to be lower to produce pronounced effects on chicken PUFA production [36]. The inclusion of 0.86 mg Se/kg feed from selenium enriched yeast in the high Se diets, gave a total Se in feed of 1.05 mg Se/kg diet. Although the high Se diet resulted in increased total fat content of the breast muscle (0.8 % in the low Se group and 0.9% in the high Se groups) compared to the low Se dietary groups, it may be too high for optimal effect on raising the level of LC PUFA. Haug et al. (2008) observed that although the there was a linear effect of increasing dietary Se level on blood Glutathion peroxidase (Gpx) levels, the efficiency (Gpx activity/Se intake) decreased as the amount of supplemented Se in the diet increased [37]. A similar effect of dietary Se level on LC PUFA production may be suggested, where an optimal increase in LC PUFA may be seen at supplemented Se levels in-between the levels used in the current study when dietary oils rich in PUFA are used.

The antiradical power (mg/g DPPH) did not differ between the dietary groups, regardless of Se level or dietary oil source, indicating that the level of both the low Se and high Se diets were high enough to sustain the measured antioxidant capacity in the chicken muscle under the current experimental conditions.

In accordance with earlier studies showing that dietary oils rich in oleic acid greatly enhance the incorporation of oleic acid into the tissues of chickens [14], the highest content of oleic acid and MUFA were found in the breast muscle of the RO containing dietary groups. The increased incorporation of dietary Se in the current study, led to higher levels of chicken breast meat MUFA levels. Zanini et al. (2003) found that increased levels of dietary antioxidants in the form of vitamin E could lead to increased deposition of MUFA present in the dietary oil, and further incorporation into meat [38]. On the other hand Hsieh e al. (2002), observed no increase in breast muscle MUFA content after increasing dietary antioxidant levels [39]. The incorporation of MUFA into chicken meat may have favorable effects on consumer health [40] as MUFA rich diets have been shown to reduce LDL oxidative susceptibility in vitro, contribute to lowering cholesterol levels [41] and prevent the conformation of atherosclerotic plaque [42]. Considering the obtained higher levels of breast muscle oleic acid and *n-3* LC PUFA, the LO, RO dietary combination may be considered as a preferable fat source to be used in chicken diets compared to the now commonly used SO.

According to The Norwegian Food Composition Table [43] the level of Se in today’s commercial chicken breast meat is 0.08 mg/kg, which is similar to the 0.1 mg/kg Se found in the meat of the low Se groups. In comparison the high Se dietary groups had 0.58 mg Se/kg breast meat, supplying the consumer with Se levels equivalent to those found in fish such as salmon (0.5 mg Se/kg) [33]. According to the Nordic Nutrient Recommendations (2004), the daily recommended minimal Se intake is 0.04 mg Se/day for women and 0.05 mg Se/day for men [44]. When eating one portion of chicken meat (175 g) [33] the low Se dietary groups would supply 0.02 mg of Se compared to the high Se which would supply the consumer with roughly 0.1 mg Se. Considering the nutritional quality of the chicken meat, the high Se level clearly improved the meat as a source of Se for the human consumer.

Interestingly the chickens consuming the two diets containing RPO showed an almost 5% lower total blood plasma cholesterol value compared to the other dietary groups. Tocotrienols, which are found in abundance in RPO, influence cholesterol synthesis by post-transcriptional suppression of hydroxy methyl glutaryl-coenzyme A reductase (HMG-CoA), the rate limiting step in endogenous cholesterol synthesis, suppressing liver cholesterol synthesis [45-47]. There are several studies showing that the positional distribution of dietary fatty acids may influence their absorption and biological effects. Compared to lard, where the palmitic acid to a large extent is found in the *sn*-2 position of the dietary triacylglyserol, the palmitic acid of PO is positioned at *sn*-1 and *sn*-3, rendering it less cholesterolaemic and atherogenic [18,48,49]. Red palm oil is rich in plant sterols which competitively block cholesterol uptake, reducing dietary cholesterol uptake and thereby contributing to reducing plasma cholesterol levels [50]. *N-3* fatty acids, both ALA and *n-3* LC PUFA, have shown to have cholesterol lowering effects. Dietary fish oil has been reported to reduce the activity of HMG-CoA reductase in rats and have an overall cholesterol lowering effect in quail [4,51,52]. The lowered cholesterol levels seen for the RPO + LO dietary groups may therefore be the result of the fatty acid compositions, the tocotrienols and plant sterol content of the dietary oils, potentially making this a good dietary oil combination for both humans and animals.

The use of vegetable oils rich in *n-3* fatty acids in chicken diets to increase the deposition of LC PUFA in tissues has lead to concerns related to the increased liability of these fatty acids to oxidation and the effect this may have on organoleptic properties. In the current study there were no differences in flavor or taste for the chicken breast meat from the 12 different dietary groups after six months of storage at -20°C. Miezeliene et al. (2011) found no effect of different levels of Se on taste or odor profiles of chicken meat and Betti et al. (2009) reported no effect on perceivable sensory characteristics when enriching a chicken diet with *n-3* rich flaxseed meal for less than 16 to 20 days [53,54]. Haug et al. (2007) found no differences in antioxidant status or organoleptic properties of meat from chickens fed low and high Se levels combined with RO and LO and stored frozen for five months [17].

## Conclusion

The purpose of the present study was to evaluate the nutritional value and sensoric properties of chicken breast meat after supplying two different levels of Se enriched yeast and six different combinations of dietary oil varying in saturated, unsaturated and *n-6*, *n-3* fatty acid compositions. In the current study, adding LO to the chicken diet resulted in a marked increase in chicken meat of ALA, *n-3* LC PUFA, including DHA, a decrease in AA and a marked decrease in n-6:n-3 fatty acid ratio regardless of accompanying dietary oil. The breast meat from the LO fed chickens may be considered to have a markedly improved nutritional value when compared to breast meat from the SO fed chickens. The addition of RO and increased dietary Se level (1 mg Se/kg feed) in the diet of the chicken both increased the level of breast muscle MUFA and oleic acid. The use of RPO in replacement for FR and PO lead to a significant reduction in chicken plasma cholesterol level in both the low Se and high Se dietary groups. Replacing animal fat with PO and RPO had no negative effects on chicken muscle nutritional value and may according to the results of the present study be good alternative fat sources for use in chicken diets in combination with linseed oil. The nutritional value of chicken meat as a source of Se and preformed EPA and DHA, should be considered when optimizing the nutritional value for chicken meat for the human consumer.

## Abbreviations

LC PUFA: Long chained polyunsaturated fatty acids; PUFA: Polyunsaturated fatty acid; MUFA: Monounsaturated fatty acid; SFA: Saturated fatty acid; EPA: 20:*5n*-*3* Eicosapentenoic acid; DPA: 22:*6n*-*3* Docosapentaenoic acid; DHA: 22:5*n*-*3* Docosahexaenoic acid; ALA: 18:3*n*-*3* Alpha-linolenic acid; LA: 18:2*n*-*6* Linoleic acid; AA: 20:4*n*-*6* Arachidonic acid; SO: Soybean oil; LO: Linseed oil; RO: Rapeseed oil; FR: Rendered animal fat; PO: Palm oil; RPO: Red palm oil; Se: Selenium; ARP: Antiradical power; DPPH: 2,2-Diphenyl-1-picrylhydrazyl; HMG-CoA: Hydroxy methyl glutaryl-coenzyme A reductase.

## Competing interests

The authors declare no conflict of interest. All authors of this research have no conflict of interest related with employment, consultancies, stock ownership, grants or other funding.

## Authors’ contributions

NFN participated in the design, execution and coordination of the study, performed the statistical analysis and interpretation of the data and drafted the manuscript. RR participated in the design of the study, assisted during sampling and arranging the sensory evaluation of the boiler meat, and critically revising the manuscript. MT participated in the design of the study, assisted during sampling and helped draft and revising the manuscript. AH participated in the design, execution and coordination of the study, analysis and interpretation of the data, and has helped draft and revising the manuscript. All authors read and approved the final manuscript.
